# Malignant adenomyoepithelioma of the breast with contralateral homologous tumor and ductal carcinoma *in situ*: two case reports

**DOI:** 10.1097/RC9.0000000000000754

**Published:** 2026-08-07

**Authors:** Yu Mao, Linjun Xie, Yanli Zou, Hongying Che, Haomiao Lan

**Affiliations:** a Department of Thyroid and Breast Surgery, Zigong First People’s Hospital, Zigong, China; b Department of Pathology, Zigong First People’s Hospital, Zigong, China

**Keywords:** breast malignant adenomyoepithelioma, case report, combined tumor, surgical approach, treatment

## Abstract

**Introduction and importance:**

Malignant adenomyoepithelioma (MAME) is a rare biphasic breast tumor with no standardized management guidelines. Co-occurrence with other breast malignancies is extremely rare.

**Case introduction:**

We report two cases with long-term follow-up: a 59-year-old female with left invasive MAME and contralateral intermediate-grade ductal carcinoma *in situ*, and a 62-year-old female with bilateral MAME. Both underwent bilateral mastectomy with sentinel lymph node biopsy (SLNB).

**Clinical discussion:**

MAME may co-occur with homologous or breast malignancies of ductal origin, suggesting a possible association that requires validation in larger studies. There is no consensus on adjuvant therapy. Both cases had negative SLNBs, highlighting the need to optimize axillary management. Molecular research is needed to develop targeted therapies.

**Conclusion:**

MAME has nonspecific clinical features. Our preliminary observations suggest a possible association with concurrent breast malignancy that requires further validation.

## Introduction

Myoepithelial cells between the breast ductal epithelium and the basement membrane form the biphasic cellular component of adenomyoepithelioma (AME), first described by Hamperl in 1970^[^1^]^. This rare breast tumor mostly affects women over 50 years old, tends to recur, and a small proportion are malignant and have metastatic potential. Malignant adenomyoepithelioma (MAME) is a rare primary tumor of the breast that has epithelial and myoepithelial components^[^1^]^. No standardized chemotherapy or radiotherapy regimens exist for MAME. Some authors have proposed using adjuvant therapies commonly employed for conventional breast cancer^[^2^]^, but their efficacy remains unproven. This report analyzes two rare MAME cases with long-term follow-up to further characterize the clinical, pathological, and therapeutic features of this disease. The manuscript was written in accordance with the 2025 revised SCARE Guidelines^[^3^]^.

## Case presentation 1

In September 2020, a 59-year-old woman presented with a 1-month history of a left breast mass, which she had ignored until it enlarged. She had no comorbidities, was not taking any long-term medications, and denied smoking, alcohol consumption, or exposure to carcinogens. No family history of breast or ovarian cancer was reported, and no germline genetic testing (BRCA1/2, TP53, PALB2, PTEN) was performed at her request.

HIGHLIGHTS

- Malignant adenomyoepithelioma (MAME) is extremely rare in primary breast tumors. We report two rare phenotypes: invasive MAME with contralateral ductal carcinoma *in situ* and bilateral MAME.
- The diagnosis of MAME relies on histopathology combined with immunohistochemistry to identify the biphasic cellular pattern.
- Sentinel lymph node biopsy remains the standard for invasive breast cancer, but the extremely low axillary metastasis rate in MAME raises the question of node-sparing protocols for selected patients, a question that requires larger studies.
- Five- and nine-year disease-free survival in our cases confirms favorable outcomes for low-risk MAME after standardized surgery.
- A possible association between MAME and contralateral breast malignancies requires multicenter validation to guide clinical monitoring.

**Figure 1.**
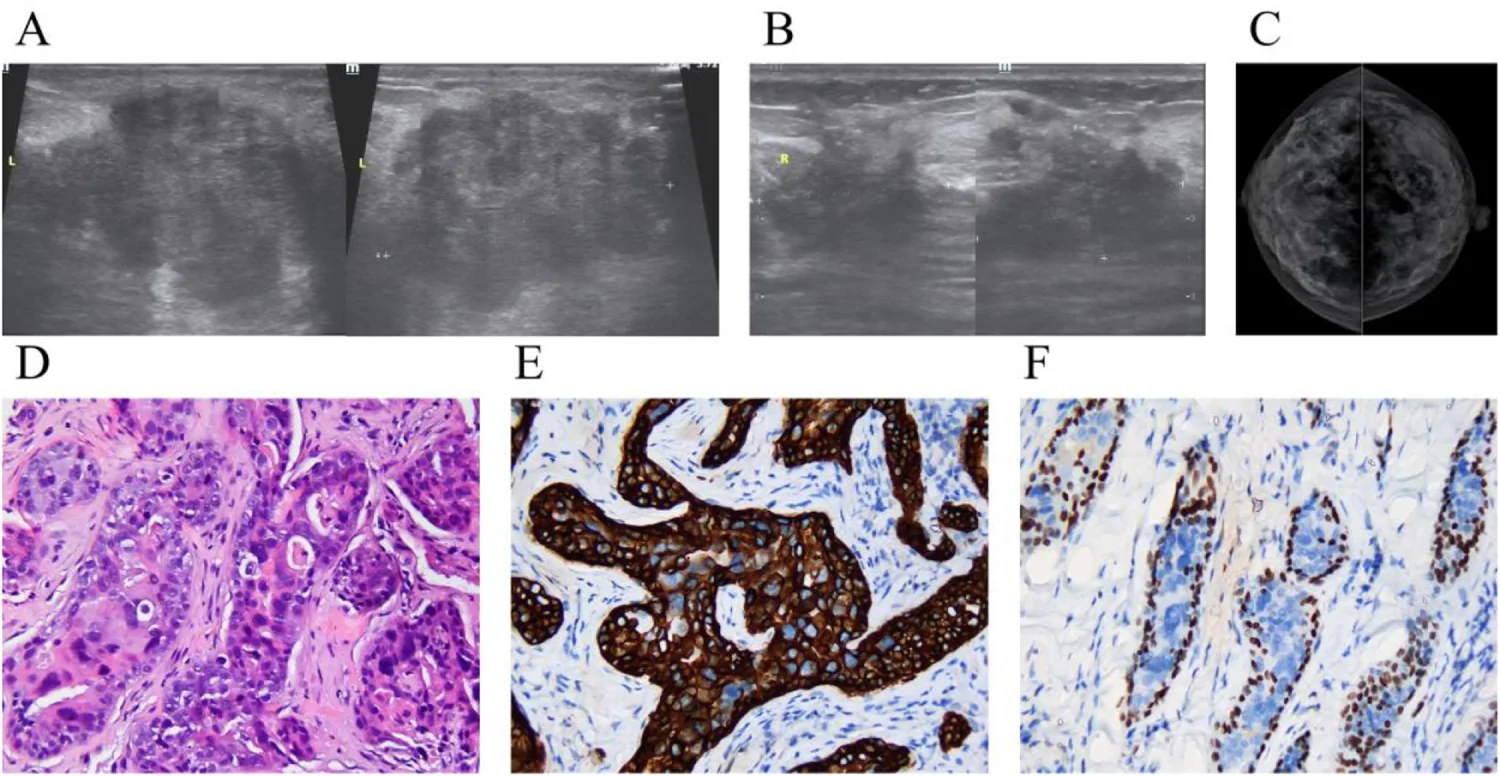
(A) Left breast ultrasound: A heterogeneous hypoechoic mass (≈4.2 × 3.7 × 2.5 cm) with an irregular, lobulated shape and internal vascularity, classified as BI-RADS 4B (moderate suspicion of malignancy). (B) Right breast ultrasound: Multiple ill-defined, irregular hypoechoic areas in the glandular layer, with no definite internal or surrounding blood flow, classified as BI-RADS 4A (low-to-intermediate suspicion of malignancy). (C) Mammography: Bilateral breast hyperplasia with heterogeneously dense parenchyma (ACR density category c), which may obscure small masses. The findings are classified as BI-RADS 0 (incomplete assessment) due to dense tissue limiting the detection of small abnormalities, requiring further imaging or follow-up. (D) H&E morphology (×400): Atypical glands arranged in solid nests with infiltrative growth are composed of glandular epithelial and myoepithelial bilayers. Glandular epithelial cells show eosinophilic cytoplasm, enlarged nuclei with coarse chromatin, marked atypia, and frequent mitoses, and the surrounding myoepithelial cells proliferate in a palisading pattern. (E) CK5/6 immunohistochemistry (×400): CK5/6 is positive in myoepithelial cells and some glandular epithelial cells. (F) p63 immunohistochemistry (×400): p63 is positive in myoepithelial cells.

Examination revealed a 5 × 4 cm hard, mobile mass in the left breast (4 o’clock, 1 cm from the nipple) and two palpable left axillary nodes; no right breast mass or supraclavicular nodes were detected. Ultrasound showed a 4.2 × 3.7 × 2.5 cm lobulated hypoechoic left breast mass (BI-RADS 4B) and multiple right breast hypoechoic areas (largest 2.7 × 2.4 × 1.7 cm, BI-RADS 4A) (Fig. 1A-B). Mammography showed dense breasts (BI-RADS 0) (Fig. 1C).

A core needle biopsy confirmed left-sided MAME and right-sided intermediate-grade ductal carcinoma *in situ* (DCIS). After a multidisciplinary discussion, the patient chose bilateral mastectomy (left modified radical, right total) with sentinel lymph node biopsy (SLNB) instead of right breast-conserving surgery, citing concerns about recurrence and psychological reassurance; she was fully counseled on all options.

Postoperative pathology: Left invasive MAME (T2N0M0, stage IIA); right intermediate-grade DCIS. 0/26 left axillary nodes and 0/6 right sentinel nodes were positive. Histology showed infiltrative biphasic nests with epithelial atypia and myoepithelial hyperplasia (Fig. 1D). Immunohistochemistry: Left MAME: ER (−), PR (−), HER2 (−), Ki-67 (20–30%), CK5/6 (epithelial+), p63 (myoepithelial+); Right DCIS: p63+, calponin+ (Fig. 1E and F).

Differential diagnoses were excluded as follows: (1) invasive ductal carcinoma, ruled out by the presence of a distinct myoepithelial layer; (2) metaplastic carcinoma, ruled out by the absence of spindle cell and squamous differentiation; (3) benign AME, ruled out by the presence of infiltrative growth, nuclear atypia, and increased mitotic activity.

Four cycles of adjuvant chemotherapy with albumin-bound paclitaxel and cyclophosphamide were administered. Standard 5-year postoperative surveillance after bilateral mastectomy was performed: clinical examination plus chest wall and bilateral axillary ultrasound every 3 months in the first 2 years, every 6 months during years 2–5, and annually thereafter. Annual systemic workup included chest and abdominal CT, a whole-body bone scan, and serum breast cancer-related tumor markers. No additional systemic therapy was given during follow-up, and no local recurrence or distant metastasis was detected at the last visit.

## Case presentation 2

In March 2017, a 62-year-old postmenopausal woman presented with a three-year history of bilateral bloody nipple discharge. Past history included right breast fibroadenoma and cyst excision. She had no comorbidities, no long-term medication use, and denied smoking or alcohol consumption. No family history of breast or ovarian cancer was reported, and no germline genetic testing was performed.

**Figure 2.**
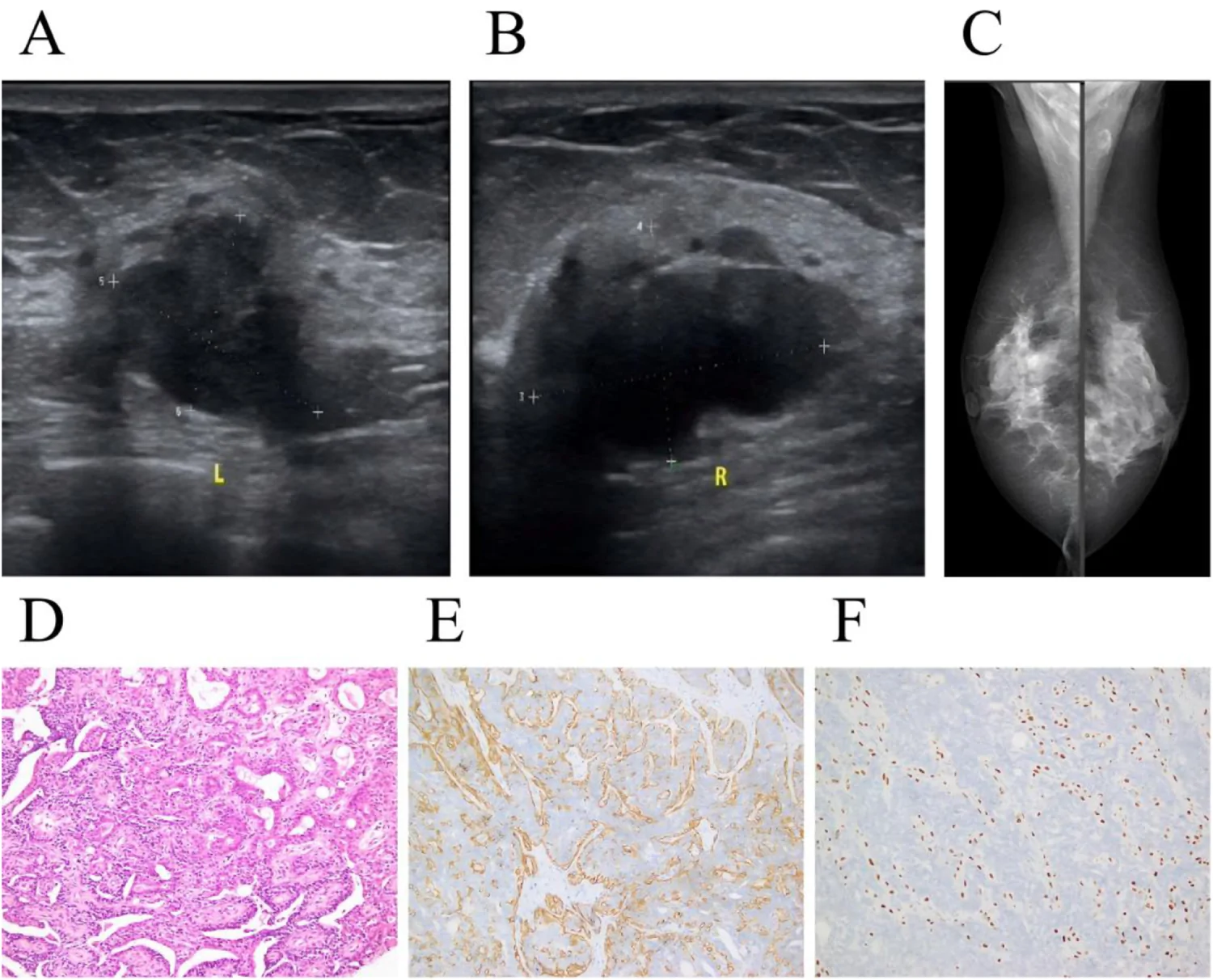
(A) B-mode ultrasound shows a hypoechoic area of approximately 1.9 × 1.2 cm in the left breast, with a relatively clear boundary and irregular shape. BI-RADS 4A. (B) A cystic lesion is present in the right breast, with an inhomogeneous echo, predominantly anechoic, measuring approximately 2.2 × 1.3 cm. A flocculent enhanced echo is visible within the anechoic area, with a relatively clear boundary and irregular shape. CDFI: No definite blood flow signals are seen within or around it. BI-RADS 4A. (C) Molybdenum target RMLO and LMLO views show bilateral breast nodules. BI-RADS 4A. (D) H&E morphology (×200): The tumor tissue exhibits infiltrative growth, composed of glandular epithelium and myoepithelium. The cytoplasm of glandular epithelial cells is stained red, with enlarged nuclei, coarse chromatin, marked atypia, and easily observable mitotic figures. The surrounding myoepithelial cells are hyperplastic, arranged in a palisading pattern. (E) p63 immunohistochemistry (×200): Myoepithelial cells are positive for p63. (F) CK5/6 immunohistochemistry (×200): Myoepithelial cells are positive for CK5/6.

Examination revealed no palpable masses; bilateral bloody nipple discharge was expressed. Ultrasound showed two left-breast hypoechoic areas (largest measuring 1.9 × 1.2 cm, BI-RADS 4A) and a right-breast cystic lesion (2.2 × 1.3 cm, BI-RADS 4A) (Fig. 2A and B). Mammography showed bilateral breast nodules (BI-RADS 4A) (Fig. 2C).

Core needle biopsy suggested left-sided invasive carcinoma, but immunohistochemistry showed discrepant myoepithelial staining. Subsequent wedge resection confirmed bilateral MAME (partially intraductal). The patient chose bilateral total mastectomy with SLNB over breast-conserving surgery due to multifocal disease and a desire to avoid long-term surveillance. She was fully informed of all options.

Postoperative pathology: bilateral MAME (pT2N0M0, stage IIA). Bilateral sentinel nodes: 0/6 positive. Histology showed infiltrative biphasic nests (Fig. 2D). Immunohistochemistry for both tumors: ER (50% positive), PR (−), HER2 (+), Ki-67 (35–45% left, 5–10% right), p63 (myoepithelial+), and CK5/6 (myoepithelial+) (Fig. 2E and F).

Differential diagnoses: (1) intraductal papilloma (ruled out by malignant cytology and infiltrative growth); (2) invasive lobular carcinoma (ruled out by E-cadherin positivity); (3) adenoid cystic carcinoma (ruled out by the absence of cribriform architecture). The adjuvant treatment regimen was gemcitabine (days 1 and 8) plus docetaxel for four cycles. The 9-year postoperative follow-up followed the same core surveillance schedule: clinical examination plus chest wall and bilateral axillary ultrasound every 3 months for the first 2 years, every 6 months from years 2–5, and annually thereafter. Annual systemic workup included chest and abdominal CT, whole-body bone scan, and serum breast cancer-related tumor markers.

Given her ER-positive status, the patient received 5 years of adjuvant endocrine therapy with 2.5 mg of letrozole daily postoperatively, with annual monitoring for treatment-related adverse effects, including bone mineral density and serum lipid profiles. No local recurrence or distant metastasis was detected at the last follow-up.

## Discussion

In the two cases reported here, Case 1 was diagnosed with invasive MAME of the left breast and contralateral intermediate-grade DCIS, and Case 2 was diagnosed with bilateral MAME.

A review of domestic and international literature shows that cases of MAME and breast cancer occurring bilaterally are rare. Contralateral breast cancer is often of the same type as MAME or is an invasive carcinoma of ductal origin. This prompts us to propose a preliminary hypothesis that there may be an association between MAME and the development of contralateral breast malignancies. However, this observation is based on a small number of cases and requires confirmation in larger, multicenter epidemiological studies. An important limitation of this study is that neither patient underwent germline genetic testing for hereditary breast cancer susceptibility genes, so we cannot exclude the possibility that the bilateral or contralateral presentation was driven by underlying germline mutations rather than a direct association with MAME itself.

Typical clinical symptoms of MAME include a mass and changes in skin color; some patients may also experience edema, nipple discharge, or nipple inversion. However, these are not specific to MAME, and diagnosis requires imaging and pathologic evaluation, particularly the identification of atypical and overly aggressive growth of myoepithelial cells.

Currently, the main treatment for MAME of the breast is surgical resection, with options such as simple mastectomy combined with sentinel lymph node biopsy, or modified radical mastectomy, depending on the type and stage. However, even with complete tumor resection with safe margins, MAME may recur and metastasize to distant sites. If distant metastasis occurs, the prognosis for patients is very poor.

Further discussion is needed about the routine use of sentinel lymph node biopsy in conventional breast cancer surgery to exclude lymph node metastasis. However, data from 15 cases of MAME summarized by the Asan Medical Center in South Korea^[^4^]^ showed that all patients underwent partial mastectomy with sentinel lymph node biopsy or total mastectomy with sentinel lymph node biopsy, and none of these patients had lymph node metastasis. Similarly, the two patients in this case report underwent total mastectomy with axillary lymph node dissection or sentinel lymph node biopsy and did not have lymph node metastasis. This finding is consistent with most reports. SLNB is currently the standard of care for staging invasive breast malignancies, including MAME. However, consistent with most published reports, both of our cases had negative sentinel lymph nodes, and axillary lymph node metastasis appears to be very rare in MAME^[^5^]^. Whether routine SLNB can be safely omitted in carefully selected low-risk MAME patients remains uncertain, and larger multicenter retrospective studies are needed to definitively address this issue.

Regarding chemotherapy and radiotherapy for MAME, due to its extremely low clinical incidence, there is currently no standardized treatment, and the domestic and international literature does not provide clear conclusions or research guidelines. Given that estrogen receptor (ER) and progesterone receptor are often negative, scholars both domestically and internationally recommend adjuvant chemotherapy or radiotherapy, and endocrine therapy may be suitable for patients with positive ER. Nevertheless, MAME is a salivary gland-type tumor of the breast and exhibits low-grade malignant behavior despite its triple-negative phenotype, which is in stark contrast to the poor prognosis of triple-negative breast tumors^[^6^]^. Therefore, the prognosis of MAME is relatively better. It should be noted that these conclusions are almost entirely based on small case series and case reports, and prognostic estimates for MAME should be interpreted with caution due to the limited available data. For MAME patients with hormone receptor-positive status, research on targeted therapy for MAME is unclear. More cases are needed to confirm the effectiveness of these drugs for MAME.

## Conclusion

In summary, MAME of the breast exhibits diverse clinical and imaging manifestations, making differential diagnosis challenging; due to its origin, MAME may be associated with the same type of MAME or invasive ductal carcinoma in the previously affected or contralateral breast, prompting consideration of the correlation between MAME and concurrent breast cancer. There is currently no clear expert consensus or guidelines on the treatment of MAME, and larger, multicenter studies are needed to establish evidence-based axillary management protocols for this rare disease. Research on the molecular mechanisms of MAME will promote the development and application of targeted therapies, and the treatment of rare diseases will continue to be expanded and improved.

## Data Availability

All data generated or analyzed during this study are included in this published article and its supplementary information files.
